# Can Universal Basic Income work for disabled people? An examination of existing UK organisational and academic positions

**DOI:** 10.1080/09687599.2023.2233688

**Published:** 2023-07-14

**Authors:** Elliott Aidan Johnson, Howard Robert Reed, Matthew Thomas Johnson

**Affiliations:** Northumbria University, Newcastle upon Tyne, UK

**Keywords:** disability organisations, disabled people, universal basic income, welfare, means-testing, conditionality

## Abstract

Universal Basic Income (UBI) has been proposed as a means of addressing a range of issues relating to welfare systems, including by removing disincentives to economic, social and physical activity. However, UK disability organisations and figures have expressed concerns about whether UBI could lead to unintended consequences for people who currently receive support conditional on needs, means and/or behaviour. In this article, we outline prominent positions regarding disabled people in the literature on UBI and welfare reforms. We find that while there are reservations about the intentions and designs of UBI, there are means of securing positive outcomes and collaboration between its supporters and disability organisations. We also attempted a consultation but were unable to obtain a significant response. This was sometimes due to an expressed inability to respond meaningfully. This serves as a call for organisations to engage with UBI as a key issue of interest to disabled people.

## Points of Interest

- Some disability organisations and leading figures have expressed concern about Universal Basic Income (UBI), a potential welfare system in which everyone receives a regular, secure and guaranteed payment. They feel that disabled people who currently receive support might lose out because they would not gain as much as others might or could even get less if existing spending is spread over the whole population.
- Some would prefer reforms that ensure disabled people receive a guaranteed minimum income that is still conditional on their income and needs.
- Some supporters of Universal Basic Income suggest that it is still possible to ensure that people with additional needs can receive more money alongside a guaranteed UBI payment.
- Most UK disability organisations appear not to have a clear (published) policy or position on Universal Basic Income.
- Disability organisations and leading figures in the movement must engage with Universal Basic Income as a policy that would impact disabled people’s lives significantly.

## Introduction

Universal Basic Income (UBI) is a system in which all permanent residents, or citizens where that is feasible, receive a regular, largely unconditional payment that ensures they have money to pay for basic needs, whether they are in work or not. It is often associated with progressive politics and the left, having been supported by, for example, the British Labour Party under Jeremy Corbyn (Labour Party 2019) and the Green Party of England and Wales (Green Party 2019). However, it has been promoted by thinkers, and even policymakers, across the political spectrum as a means of promoting rights (Pettit 2008), efficiency (Gordon 2014), growth (Sheahen 2012) and supporting flexibility in the labour market (Harrop and Tait 2017). Indeed, even free-market economic theorist Milton Friedman, an inspirational figure for a number of prominent conservative politicians (Laidler 2007), promoted a negative income tax to Richard Nixon who, as President, presented a bill that failed to gain traction (Kay 2017). Negative income tax is a system in which people whose assessed income falls below a certain level receive a payment from the state rather than paying income tax to it (Sheahen 2012, 3-4). Unlike UBI, therefore, it retains means testing. Criticism of UBI has equally emerged from right, centre and left, including UK trade unions (Henderson and Quiggin 2019). There are now well over 100 pilots or ‘micro-pilots’ of schemes similar to basic income around the world, with a range of positive outcomes noted (Stanford Basic Income Lab 2020). Proposals for the first micro-pilots in England – in Jarrow, South Tyneside, and East Finchley, London – were recently proposed by some of the authors of the current paper (Johnson, Goodman, et al. 2023).

Elsewhere, we have argued for UBI on the basis that it has the potential to act as a socioeconomic upstream intervention to affect social determinants and improve both mental and physical health outcomes (Johnson et al. 2022). Despite this prospective impact as well as the vulnerability of disabled people to welfare reform, there has been limited, often ad hoc, research on the effects of UBI on disabled people. Such research is crucial. In this paper, we employ, broadly, the social model of disability (Shakespeare 2017) which emphasises that it is the organisation of society that disables people who have impairments, whether, for example, physical, mental, sensory or cognitive in nature.

According to a measure that is consistent with the core definition of the UK’s Equality Act 2010 definition, disabled people comprise 21% of working-age people and 22% overall (Department for Work and Pensions 2022, Table 4.1). They also face a range of intersectional determinants (Rhode et al. 2012; Activity Alliance, and IFF Research 2020, 118-123), with substantial overlap between disabled people, people with long-term and multiple health conditions – including stress-related conditions (Rhode et al. 2012) – and people with lower socioeconomic status (SES). This includes much higher rates of poverty, with a gap of 21 percentage points between the rates for disabled (38%) and non-disabled (17%) working-age people in UK 2019/20 data (Joseph Rowntree Foundation 2022, 58). Disabled people are also often subject to particular health needs and effects in relation to social policy (Johnson and Spring 2018) and are disproportionately affected by welfare and reforms to welfare systems (Johnson and Nettle 2020). Importantly, given the ‘universal’ nature of UBI, disabled people face estimated additional costs of £583 per month on average to have the same standard of living as their non-disabled peers (John, Thomas, and Touchet 2019).

The existing UK system of needs and means tested benefits was substantially overhauled during the period of austerity politics that followed the global financial crisis of 2007-2008 (Johnson and Nettle 2020). A narrative of ‘deserving vs undeserving’ welfare recipients was deployed, most effectively (but not solely) by Conservative-led administrations from 2010, in support of greater conditionality and a redrawing of the criteria used to judge needs that resulted in reduced eligibility, in particular with regard to mobility (Johnson and Nettle 2020). The reformed system included, among others: Employment and Support Allowance (ESA), which the New Labour Government introduced alongside the Work Capability Assessment in 2008 to replace Incapacity Benefit as the primary means-tested form of income support for sick and disabled people (Kennedy et al. 2019); Universal Credit, introduced by the Conservative-led Coalition Government and rolled out from 2013 to integrate a large number of separate welfare payments including the main non sickness- or disability-related unemployment benefit, Jobseeker’s Allowance (Welfare Reform Act 2012); and Personal Independence Payment (PIP), which began to replace Disability Living Allowance (DLA) as the most prominent non-means tested support for the additional costs faced by disabled people at the same time as the Universal Credit rollout (Welfare Reform Act 2012). There has since been a further integration of income-based ESA into Universal Credit (Department for Work and Pensions 2022). Despite this large-scale and ongoing reform, with an intention to scrap the Work Capability Assessment recently announced (Kennedy and Hobson 2023), it is difficult to argue that the Government met any of its substantive explicit or implicit objectives (Johnson and Nettle 2020), with some subsequent revisions, such as the effective removal of reassessments for people with severe, lifelong conditions, marking a partial return to the previous approach to administration of disability benefits (Johnson and Nettle 2022).

Ironically, the stated intentions underpinning the introduction of Universal Credit, namely, to streamline a complex system and remove disincentives to work by ensuring smooth transitions on and off payments when recipients breached income thresholds (Department for Work and Pensions 2010), are shared with those of many of basic income’s proponents. Unfortunately, a higher than initially intended taper rate – the amount of the benefit withdrawn as income increases – of 65% (only amended much later to 63% then finally to the originally planned 55%) resulted in Universal Credit recipients receiving much smaller financial benefits from taking up low-paid work (Finch and Gardiner 2018; Brewer and De Agostini 2015). An extended wait period and delays in assessment before payments were made as well as a regime of strict sanctioning for failing to meet benefit conditions also meant that many were left without income security (Wright and Dwyer 2022). Indeed, the United Nations’ Special Rapporteur on Extreme Poverty and Human Rights described the system as ‘fast falling into Universal Discredit’ (Alston 2019, 12).

Following this period of extreme conditionality, however, there appears to have been a very significant shift in public perceptions in the wake of the COVID-19 pandemic and subsequent cost-of-living crisis, in which a large proportion of the population required financial support from the state. Unconditional welfare systems such as UBI now receive very high favourability rating among a large majority of the population (e.g. Johnson, Johnson, and Nettle 2022). Although the UK Government has largely ignored this shift with regard to policymaking, devolved administrations have taken the opportunity to pursue alternatives within their powers. For example, the Labour-led Welsh Government (2022) has introduced a pilot of basic income among care leavers and the Scottish Government a reformed system of disability payments (Scottish Government 2023) as well as plans for a Minimum Income Guarantee (Minimum Income Guarantee Expert Group 2023), which provides an income floor below which people cannot fall but differs from UBI in being means tested.

Despite the issues identified with the current system of benefits conditional on needs, means and/or behaviour, some disabled people’s organisations have taken a public position against the concept of UBI in the believe that it may, in reality, lead to a relative reduction in disabled people’s financial security and an increase in exclusion from work (Disabled People Against Cuts (2019). In common with proponents of UBI who have sought to address these concerns (Duffy and Elder-Woodward 2019), we have outlined means of advancing a universal system which also contains supplements for particular needs, such as those required by disabled people. These schemes included a lower, introductory scheme and an intermediate scheme which would be provided in addition to all current conditional forms of welfare. Importantly, we also included a full Minimum Income Standard (MIS) scheme that would replace most means-tested benefits (apart from those that vary significantly such as housing benefit) while leaving open options for retaining and reforming or replacing needs-tested benefits for disabled people (see Reed et al. 2023). The project within which these schemes were developed sought consultation with disability organisations and leading figures between 8 and 31 August 2022 through either an online survey, an accessible Word document questionnaire or an interview *via* Microsoft Teams to evaluate and address issues within those schemes. However, of 18 organisations and figures approached, who were selected by the project team based on their relevance to the subject being examined, just four took part, three by Word questionnaire and one by online survey with informed consent taken at the start of each. Three of the non-respondents stated that they lacked expertise or a position on the policy and the remainder either did not take part after initially indicating that they would, were unable to take part before the (extended) deadline or did not respond at all after multiple invitations. Given the small number of participants, we present relatively detailed individual responses, with some thematic grouping where possible.

This article takes that absence of formal engagement and seeks to set out the pressing reasons for serious examination by disability organisations and figures within the movement. We identify, analyse and categorise those concerns that have been formalised by disability organisations and figures regarding implementation of UBI in order to codify a set of tests for formulation of schemes. This enables us to produce preliminary evaluation of three schemes developed for public health purposes, providing a platform for reconsideration of UBI by advocacy bodies. We start by setting out those positions that have been expressed in the literature.

## Existing positions in the literature

One argument advanced by supporters of UBI is that it has, if carefully configured, the potential to free disabled people from work capability assessments and error-prone decision making. They claim it would avoid under-claiming of welfare and enable everyone in society to be pursue their own flourishing without harmful disincentives, even if some sort of assessment of additional needs were to remain (Standing 2019, 19, 26, 35, 36, 63, 73). However, some disability organisations have indicated their opposition to UBI for a range of reasons.

Disabled People Against Cuts (2019) argues that the ‘bad idea’ and ‘unworkable policy’ (2019, 4, 12) would likely be pursued on the basis of fiscal neutrality, or at least fiscal constraint, reminiscent of the ‘widespread harm’ under the guise of ‘progressive ideas’ effected by Universal Credit. This, they contend, would mean that currently targeted welfare payments would be diluted and spread across the entire population. This would lead to a decrease for most disabled people through increased restriction in eligibility for additional payments and pressure on social care funding (Disabled People Against Cuts 2019, 14-15). They also argue that the need to continue having assessment to meet additional needs means that claims ‘that a UBI would end such testing and the associated stigmatisation are overstated’ (Disabled People Against Cuts 2019, 14-15). Finally, they suggest that ‘UBI adapts to a flexible model of employment which favours employers while further disadvantaging disabled workers’ (Disabled People Against Cuts 2019, 14-15).

Participants involved in a ‘pop-up think tank’ undertaken by Inclusion Scotland (2020, 21) that brought together ‘disabled people and others from across a range of sectors for meaningful, focussed and strategic-level discussion’ echoed these concerns but considered inclusion of disabled people in work on UBI as crucial. They believed it essential to ‘ensure that disabled people not be avoided because their participation represented something that was “too difficult” to achieve under the current system, due to the administrative challenges of moving disabled people between systems’.

The Scottish Campaign on Rights to Social Security (SCoRSS) reiterates concerns about a net loss in welfare assistance for disabled people and argues that it is essential that any changes to disability assistance be future proofed to ensure that it would work well under a ‘Citizens Basic Income’ and be ‘well-connected to access to other public services for disabled people’ (2020, 29). Importantly, the report highlights that ‘SCoRSS members believe that regardless of what wider changes are made to other social security entitlements, a separate non-means tested social security payment for disabled people should be maintained’ (SCoRSS 2020, 30).

The Commission on Social Security is an independent group led by Experts by Experience, including several disabled activists and academics, supported by funding from the charity Trust for London. It undertook two large-scale consultations on how social security should be structured. Its proposals include several components. First, there would be a Guaranteed Decent Income (GDI) equal to MIS for any applicants aged 18+ irrespective of work status. However, it would be tapered through a tax rate of 45p in each £1 earned above an allowance of £512 per month, which would be assessed through a ‘light touch tax self-assessment approach’ (Commission on Social Security 2022a, 2). It is unclear how often this self-assessment would take place and the plans include scope for ‘voluntary’ deductions from GDI, but GDI as a whole would be unavailable to those with savings of more than £85,000. The GDI approach more closely resembles a negative income tax approach than a Basic Income Guarantee with unconditional payments (Sheahen 2012, 3-4). There would be an as-yet undecided Disability Supplement to GDI, a Child Benefit (at £50 per child per week) (Commission on Social Security 2022a, 5) and a new disability benefit that would not be treated as income in relation to GDI. Housing costs would be dealt with separately, with a longer-term aim of large-scale building of social housing (Commission on Social Security 2022a, 3).

The new disability benefit would be non-means-tested, like Disability Living Allowance and Personal Independence Payment, and would be based on the social model of disability and designed in full co-production with paid Deaf and disabled people (Commission on Social Security 2022a, 6). Principles include annual uprating, no one financially worse off, minimal burden on claimants, award based on need with no targets and no risk of people being left with nothing at any point (Commission on Social Security 2022a, 6). They propose that there would be a comprehensive range of areas for support considered, with individualised assessments based on the needs the claimant identifies, a collaborative approach to decision making with assessors and decision makers trained to have detailed knowledge of the social model of disability and other areas of knowledge required (Commission on Social Security 2022a, 7). Decisions would be paper based where possible with full accessibility, transparency and rapid appeals (Commission on Social Security 2022a, 7). There would also be lifetime awards available and longer gaps between reviews, with the lowest rate equivalent to the PIP standard rate for daily living and mobility, the middle rate the current PIP enhanced amounts and the higher rate £1,000 per month to match Scope’s finding that 1 in 5 disabled adults and nearly one quarter of families with a disabled child face extra costs of over £1,000 per month (Commission on Social Security 2022a, 7). The Commission also proposes reforms in a range of other areas such as job conditions, education, health and care, along with a progressive local tax (Commission on Social Security 2022a, 8).

The Commission report only mentions UBI on that basis that proposals have failed to meet its first principle: ‘Make sure everyone has enough money to live – and support extra costs e.g. to do with disability and children’ (Commission on Social Security 2022b, 36). The reference the highest UBI proposal as having been the RSA’s £92 per week.

Other disability researchers and organisations have been far more positive about the prospect of UBI and its impact on disabled people. Building on their previous collaborative work (Elder-Woodward and Duffy 2018), Duffy and Elder-Woodward (2019, 19) discuss the compatibility between UBI and the Independent Living Movement and seek to resolve some of the issues identified above. They describe a system of UBI+, in which additional payments remain to address people’s additional needs. They claim the ‘extra income supplements would be introduced in accordance with the spirit of UBI’ (Duffy and Elder-Woodward 2019, 20), with no means testing, spending conditions or behavioural conditions, which have been found to have particularly harmful effects on disabled people’s activity (Activity Alliance, and IFF Research 2020; Johnson and Spring 2018).

With regard to assessment or claims process, Duffy and Elder-Woodward suggest it ‘should be designed with disabled people to be empowering and respectful. Obviously, this would be radically different from the medical and professionalised models of assessment currently being used’ (2019, 20). They point to the benefits that UBI + could have for disabled people, including highlighting better physical and mental health, and reduction in poverty, particularly given their higher rates among disabled people (Duffy and Elder-Woodward 2019, 20). They also emphasise the reduction in income insecurity it would provide given that systems like what is now a component of Universal Credit ‘are organised so that the whole of your income is dependent on proving the negative impact of your impairment on your ability to work’ (Duffy and Elder-Woodward 2019, 21-22). They contend that the process is inherently stressful and made worse by a drawn-out appeal system that leaves individuals challenging a decision without income until it concludes (Duffy and Elder-Woodward 2019, 21). They argue that a key benefit is to remove the poverty trap created by means- and needs-testing and behavioural conditionality that means people with fluctuating conditions cannot take on work straightforwardly in the periods during which they are more able to do so (Duffy and Elder-Woodward 2019, 21). They claim that UBI + achieves a significant aim of the Disabled People’s Independent Living movement by putting disabled people in control of funding their own care and support. Finally, and in common with our suggestions (Johnson and Nettle 2020), establishing a welfare system common to all citizens means that there is political reason to ensure the benefit is kept at a level to ensure support among the population (Duffy and Elder-Woodward 2019, 21).

The authors also identify the key reasons for opposition to UBI among some in the Disabled People’s Independent Living movement. This includes those outlined by Disabled People Against Cuts (2019) above, founded in a fear that UBI is supported by some neoliberals who would inevitably seek as low a UBI as possible primarily for the benefit of employers and as part of a long-term trend of excluding disabled people both from welfare and employment as labour costs are driven down through broader automation efforts (Duffy and Elder-Woodward 2019, 23). In particular, they cite Martinelli’s (2017) claim that the ‘unavoidable reality is that such schemes either have unacceptable distributional consequences or they simply cost too much’. The authors consider that some of this criticism is founded on a belief within the Independent Living movement that being employed in the paid workforce is a crucial component of inclusion (Duffy and Elder-Woodward 2019, 23). Instead, they paraphrase Lyons (2019, 4) and claim that we ‘are at a crossroads and we face a choice between a capitalist or a human conception of life’s social value’ (Duffy and Elder-Woodward 2019, 23). They further highlight an argument we have made (Johnson and Johnson 2019, 263), that UBI has the potential to tip the balance in favour of workers, providing a safety net to turn down ‘bad work’ and negotiate better, more meaningful and dignified contractual obligations.

Crucially, they identify two potential groups of disabled people for whom each side of the argument may resonate. The first are those who may benefit from the status and resources of suitably adapted, legally protected employment in the current system and those who ‘can make a vital contribution to community life through caring for people, or for the commons, through civic and political action or through artistic endeavour’ (Duffy and Elder-Woodward 2019, 23).

In terms of creating a ‘partnership’ between UBI proponents and disabled people in the Independent Living movement, the authors suggest that piloting UBI + through conversion of existing benefits *via* small changes in conditions could be a feasible first step (Duffy and Elder-Woodward 2019, 24). They also argue that UBI proponents need to avoid claiming that UBI is a ‘panacea for solving every social problem nor for meeting every vital need’ and propose universal public services as an essential additional component of reforms (Duffy and Elder-Woodward 2019, 24). They summarise their view that it would

benefit the UBI movement to adopt this vision and to see the fight for UBI as part of an effort to build an emancipatory welfare state. It is not enough to think in terms of meeting needs, instead we need to see the purpose of the welfare state as being to empower potential (Duffy and Elder-Woodward 2019, 25)

Richardson and Duffy (2020) provide specific detail on UBI plus bolt-ons using MIS data to cover individual, home, travel and activity costs as well as those associated with long-term illness, disability or caring.

## Our proposals

Our proposals (Reed et al. 2023) overlap substantially with those of Duffy and Elder-Woodward. We have proposed that UBI at MIS level would replace almost all *means*-tested benefits apart from Universal Credit elements covering childcare and housing as well as Housing Benefit for pensioners. Contributory benefits that replace income, like New Style Employment and Support Allowance and Jobseeker’s Allowance would also be replaced by the MIS-level UBI. However, reformed needs-based benefits (currently Personal Independence Payment [PIP] and Disability Living Allowance [DLA]), would remain in some revised form (Reed et al. 2023). Under the lower schemes, means-tested benefits would remain. This is not an endorsement of the current system of needs-testing, but, rather, it would ensure that UBI’s simplicity of administration would be retained while preventing disabled people from losing out relatively from the new system. Given differing needs in areas such as care, transport and housing, a system along the lines of a UBI + was felt to be both the fairest and most feasible means of creating a UBI policy that could be introduced in advance of expansion of public services and housing.

In shaping the policy with an aim to benefit the mental health of young people, we outlined three UBI schemes and presented them to 28 14- to 22-year-olds in Bradford, West Yorkshire, through online surveys and citizen engagement workshops to understand which features might best meet their needs (Johnson, Webster, et al. 2023). Disabled young people formed a substantial proportion of participants in both the workshops and the online surveys from which they were drawn, with a quarter of workshop participants being disabled. The three schemes they reviewed (for full details see Reed et al. 2023) ranged from a starter scheme of an unconditional £41 per child, £63 per adult aged 18-64 and £190 per adult aged 65+ supported by the vast majority of the current conditional system, to a full Minimum Income Standard-level scheme of £95 per child; £230 per adult aged 18-64; £230 per adult aged 65+ replacing all benefits except for those where needs differ significantly between individuals, namely needs-based disability support, housing and childcare. There was also an intermediate scheme with features somewhere between the two. In the three schemes considered, income tax and National Insurance contributions (an employment-based tax) were also revised. We set out the highly progressive distributional effects of these schemes, arguing that the more expensive schemes are also more likely to produce significant returns on investment in terms of health, economic and social benefits (Johnson et al. 2021; Reed et al. 2023).

## Consultation and areas of consensus and disagreement

The core principle that emerges from all voices on disability within UBI is that disabled people must not lose out from a move to a new system. With regard to non-means-tested disability benefits, this principle, as well as fiscal reality, indicates that it would not be desirable to raise UBI to an amount that would cover costs of disability among the entire (including non-disabled) population. The four respondents to our consultation were relatively unified in thinking that a payment that would cover the cost of disability for everyone would not work. One respondent from an organisation with an interest in UBI and how welfare systems affect disabled people said that while he believed a generous UBI would help a range of disabled people, including those who have been ‘deprived of’ disability-related benefits, we ‘should not be so afraid of assessment as to try and capture all extra needs in this way’. He believed that this would be both ‘fiscally unrealistic’ and ‘disrespectful to the reality of disability’. He suggested that ‘we should tackle the confusing and largely disrespectful array of assessment services head on’ and that ‘ending poverty and meeting the extra costs caused by disability are simply not the same challenges and muddling them up probably will undermine both efforts’. An academic respondent said that the prospect of a UBI covering the additional costs of disability for all, including non-disabled people, ‘is a terrible idea – it does not contribute to creating a more equal society between disabled and non-disabled people (because many disabled people will still face additional costs)’.

Instead, all respondents supported the concept of a substantially reformed system of needs-based payments. One academic respondent said that in addition to the assessment process being ‘completely overhauled’ with regard to needs- and means-tested benefits, that it is ‘very easy to say “the assessment should be better”, and much harder to specify what this means; few disabled people’s organisations or disability charities have tried this’. He also cautioned that

it’s a real problem to just assume that this is a simple task – this is one of the problems with Universal Credit, where the people that came up with the idea just didn’t think about how the system would work for disabled people, assuming that things like assessments were technical issues that other people could solve further down the line.

Given these issues, a reformed system of conditional payments to cover costs related to disability, illness and incapacity seems both sensible and inevitable. Duffy & Elder-Woodward’s (2019) and Richardson and Duffy (2020) proposals as well as those of the Commission on Social Security (2022a, 2022b) on this front are, therefore, a good starting point. Needs-based supplements are the most practicable and ethically justifiable means of taking account of the additional costs of living, whether administered within a UBI supplement or separately alongside other additional needs, e.g. housing, childcare etc. The additional payment on top of a Guaranteed Decent Income, as the Commission on Social Security (2022a) proposes, would appear to duplicate mechanisms of support for additional living costs of disability. Benefits such as disability- and health-condition-based Universal Credit are intended to replace income that would otherwise come from work. In a MIS-level UBI scheme, dealing with this solely through a non-means-tested system would appear sensible. Indeed, the Government announced in the 2023 Spring Budget (HM Treasury 2023) that the Work Capability Assessment would be abolished, instead assessing need only through the PIP assessment. This means that our MIS-level UBI would avoid reintroducing an instrument of means- and needs-testing that is soon to be removed.

Our proposals also address some of the issues raised by the Commission on Social Security (2022b, 36) in relation to UBI payments being too small to meet the requirements outlined by the MIS. In Scheme 1, this would be through the existing conditional system, but Scheme 2, in itself, approaches the Guaranteed Decent Income even without conditional support, while Scheme 3 exceeds the GDI significantly in most cases. Crucially, under UBI, everyone would receive the whole sum without claiming, without assessments and without a taper over a certain earnings level. Schemes 1 and 2 are compatible with the kind of continued assessment that the Commission proposes, as, for the purposes of modelling, Universal Credit (which has its own taper rate) along with other means-tested benefits are retained. Scheme 3 would also ensure that disabled people were never left without any income, regardless of needs-tested supplements, and that they would remain relative net beneficiaries of the welfare system compared with non-disabled people due to the needs-tested component. In the event that schemes 1 or 2 were implemented and Work Capability Assessments were retained (or reintroduced given the Government’s Spring Budget announcement) in some form, Ben Geiger’s (2018) report recommendations, focusing on transparency, lived experience, evidence, expertise, accuracy, reduced conditionality, trust and co-production, provide further specific suggestions for producing a system that reflects the needs of disabled people. Indeed, even in Scheme 3, such principles may be important in reforming assessment of need.

When asked about replacement of needs-based cash benefits currently received by disabled people with services, respondents were opposed due to reduction in autonomy and choice, which reflects the disability rights movement’s position overall. One academic who responded stated that the prospect

is a terrible idea! Universal services are great in all sorts of ways. But DLA/PIP/AA are relatively unique benefits internationally in giving people cash to decide the best way of meeting their needs, to cope with the additional costs they face. This is autonomy-enhancing. Replacing this with services (that a ‘benevolent technocrat’ determines, whether or not this is in consultation with claimants themselves) would be disempowering.

Another academic said that he ‘would want to retain needs-based benefits, and have a fair and transparent system for allocation. I still think you need an assessment. But it should not be punitive as at present’. A representative of a disability rights organisation said that it

would remove peoples’ choice and control over how they spend payments so we wouldn’t support it. Of course many disabled people who need social care now have to pay most or all of their PIP, DLA [care components] and AA in care charges which is a major factor in pushing disabled people into poverty so it is essential that social care is free at the point of need and not charged for. At the moment in England, Hammersmith and Fulham is the only LA where social care is free.

The respondent from an organisation with an interest in UBI and how welfare systems affect disabled people echoed these concerns, highlighting the flexibility and autonomy that cash-based benefits bring and that replacing cash with goods is ‘likely to be highly inefficient and frustrating’, with additional autonomy-reducing assessment for services. Importantly, he asked why disabled people alone should ‘face this extra burden of inflexibility’, and that any case for replacing cash with services should be largely universal with an aim to ‘normalise and simplify’, unless there is good reason for an alternative. He further highlighted that the development in social care has been from service to Personal Budget on the basis of disability rights campaigning. Given that a core aim of UBI is to enhance autonomy, it is essential that disabled people are not disempowered through such reforms. The responses indicate that there may be scope to replace portions of UBI with services where disabled people are not specifically singled out and where better outcomes for users are possible, but that cash transfers should be the default.

With regard to Carer’s Allowance – which is a benefit in 2022/23 of £69.70 per week for people providing at least 35 h of care with earnings of £132 or less after tax, National Insurance and expenses (Government Digital Service, 2023) – the three respondents who provided an answer were unified that the current level of support is inadequate and that a much larger rate of remuneration is required. The respondent from an organisation with an interest in UBI and how welfare systems affect disabled people highlighted that UBI ‘can help people, families and communities provide informal unpaid support to each other. This also includes the ability of disabled people to contribute to their community in any way they wish without risking their benefits’. He argues that this may actually help to reduce costs elsewhere and that this needs to be examined further. He also suggests that many ‘disabled people may be understandably nervous that a presumption that their needs can be met by family or friends will become common place if UBI levels free up more voluntary time’. He states that there is a need to find an ‘intentional balance’ between commodifying time (e.g. under current wage economy systems) and decommodifying it (e.g. such as those that include UBI) so that payment for professional services is possible while also ‘giving people more time to do things that they value – including doing things of social value’.

Two of the three respondents who commented on whether means-tested benefits should be retained (as in schemes 1 and 2) or replaced with a common UBI payment (as in Scheme 3) said that they favoured replacement. One of the academic respondents said that there are

Advantages for disabled people in getting what everyone gets, and everyone getting something, to remove stigma of benefits for disabled people. If PIP truly meets additional costs, then [it] should not be associated with poverty. [There is a] need to invest in proper vocational rehabilitation and return to work.

The respondent from an organisation with an interest in UBI and how welfare systems affect disabled people said that he is opposed to means-tested benefits because they add to ‘the burden of relative poverty’ further disincentives to ‘earn,… to save, marry, take risks, gain skills or to be reassessed’ as well placing those in receipt of social security in an outgroup that tends to encourage ‘othering, stigma and political scapegoating’. The respondent who represents a disability rights organisation disagreed with the removal of means-tested benefits, arguing that ‘the amounts suggested would be too low in most cases to make any real difference to poverty levels especially given the massive inflation rates we’re now facing’. Contrasting with this conclusion is microsimulation modelling (Reed et al. 2023) that has shown that all three schemes would have a very substantial impact on poverty rates as a whole. However, it did not examine households or benefit units with a disabled person specifically. Regardless, Scheme 3 would effectively eliminate relative poverty based on current median salary (rather than the adjusted one following the scheme’s introduction). This does not mean, however, that the material deprivation would not continue to be felt by some households with larger expenses, for example often those that include a disabled person.

Regarding favoured schemes of the three presented, two of the three respondents who provided an answer indicated a preference for Scheme 3, with the other choosing Scheme 2. Two of the three, an academic and one from an organisation with an interest in UBI and how welfare systems affect disabled people said they would feel able to campaign for the introduction of UBI based on their favoured scheme (2 and 3 respectively), but the respondent who represents a disability rights organisation said that their organisation favoured supported a Minimum Income Guarantee as outlined in the Commission on Social Security’s (2022b) report. The remaining academic respondent said that they had concerns about UBI because

they have political dynamics that lead to nativism – i.e. ‘this is money you get for being British, so we’re going to restrict migrants access to it’. Indeed, contributory benefits (like ESA/JSA) have been more generous to migrants than needs-based benefits have been.

While UBI is sometimes promoted as a ‘citizens’ income’, in a UK context, it is unlikely that UBI could be restricted to British citizens only. This is because there is no national database of citizens or mandatory ID card system. Instead, it would likely be paid to citizens and permanent residents, the latter of whom retain non-UK citizenship. Even if solely due to pragmatism, as a UBI would have to be provided efficiently given its scale, there are reasons to believe that there could not be a nativist element in the way the respondent contends. Indeed, ESA and JSA have through their histories had both contributory and means-tested versions, with the least well off likely to be applying to the latter. As it relates to disabled people, and in line with one of the respondent’s views, there is good reason to think that a welfare system that covers the whole population will lead to a reduction in ‘othering’ and stigma (Johnson and Nettle 2020).

In terms of anything else that could be done to make UBI work better for disabled people, the respondent from an organisation with an interest in UBI and how welfare systems affect disabled people a said that

as it stands I think Scheme 3 doesn’t unite the disability and anti-poverty communities and by taking a conservative stance on the current mess of disability benefits – and even the relationship to care services I think an opportunity is being missed.

The respondent from a disability rights organisation felt that ‘a neo-liberal financial framework is unlikely to ever value disabled people sufficiently’. There are challenges in this position based on alternative options. The Minimum Income Guarantee that the respondent’s organisation favours similarly resides within the existing UK market economy, with that system potentially replicating existing forms of welfare that supplement the National Minimum Wage and National Living Wage with means-tested benefits. UBI, on the other hand, would be paid regardless of income and there is good reason to suggest that it would put employees in a stronger bargaining position with employers as it would provide them with the ‘power to say no’ to unreasonable demands or insufficient salaries (Johnson and Johnson 2019). For disabled people, it would guarantee a replacement of means-tested benefits with an unconditional payment, simplifying the process for taking on short-term or part-time work if they feel that such a pursuit is possible and beneficial.

## Discussion

Through this examination of the literature and small consultation, we have found that while a diversity of opinions among disability organisations and researchers exists, including some who strongly oppose its introduction based on previous formulations, there appear to be few or no insurmountable practical challenges to the development of a UBI policy that addresses concerns and has the potential to secure broad agreement, if not consensus.

In particular, it is possible to account for differences in needs within or alongside a UBI payment. This would also ensure that disabled people are not left relatively worse off. Here, it is essential to note that from April 2013 to October 2021, 25% of former working-age DLA recipients who registered for reassessment under PIP have been left without any disability-based support in the move from Disability Living Allowance to Personal Independence Payment (Department for Work and Pensions 2021). This does not include DLA recipients who did not register for reassessment. There is no possible situation in which these disabled people would have been left without any income under a UBI, even if it were not the ideal amount to cover costs. In addition, there are broader benefits from a welfare system that focuses on the needs of the whole population rather than a specific group (Johnson and Johnson 2019; Johnson, Aidan Johnson, et al. 2023). We have previously argued that one of the key means of ensuring that welfare recipients are no longer viewed as an undeserving outgroup is to ensure that the system is seen to benefit a far greater group of people, as Child Benefit did previously (Johnson and Nettle 2020; Johnson, Johnson, and Nettle 2022; Johnson, Aidan Johnson, et al. 2023).

On the other hand, the Commission on Social Security’s (2022a 2022b) proposals run the risk of retaining or even increasing the complexity that exists within the current system while failing to increase incomes among claimants substantially or reduce opposition to welfare among the large proportion of the population that is unlikely to benefit materially. However, we believe that a blend of the Commission’s proposals on needs-based payments, Duffy & Elder-Woodward’s UBI + and Richardson & Duffy’s more specific proposals based on MIS have the capacity to address key challenges both with the current system that is conditional on means, needs and behaviour as well as with proposed UBI schemes.

The low level of response to the consultation indicates substantial challenges in pursuing reform of both the existing system and the introduction of new ones that are informed by participatory methods. Organisations and campaigners who argue that existing systems are inadequate must be willing to commit resource, even if just in time, to support positive efforts to address the issues they identify. It is important that individuals within organisations feel empowered to participate in consultation activities even when organisational policy has not yet been settled. Indeed, it is an essential component of developing such policy and representing stakeholders effectively.
